# Identification of a novel interaction between the M2 proton channel of influenza A virus and cyclin D3: consequences for cell cycle progression

**DOI:** 10.1186/1753-6561-5-S1-P70

**Published:** 2011-01-10

**Authors:** Y Zhang, F Kien, HL Ma, Jane Tse, LLM Poon, B Nal

**Affiliations:** 1 HKU-Pasteur Research Centre, Hong Kong, Hong Kong SAR; 2 Department of Microbiology, Li Ka Shing Faculty of Medicine, The University of Hong Kong, Hong Kong SAR; 3 Department of Anatomy, Li Ka Shing Faculty of Medicine, The University of Hong Kong, Hong Kong SAR

## 

To define cellular factors involved in the life cycle of influenza virus, we have used a genome-wide yeast-two-hybrid screening approach. Interestingly, we have identified a novel interaction between the 54 amino-acid cytosolic tail of influenza A virus M2 proton channel protein and the cyclin D3 cellular protein, a key regulator of the cell cycle G1-S transition. Very little is known about crosstalks between influenza A virus and the cellular machineries that regulate the cell cycle.

We confirmed the physical interaction between M2 and Cyclin D3, by co-immunoprecipitation studies in human epithelial cells. We then conducted immunofluorescence assays to analyze the relative distribution of cyclin D3 and M2 in infected A549 lung epithelial cells. Interestingly, we observed that cyclin D3 is relocated to or retained at the Golgi apparatus, where M2 is concentrated, at 5 hours post infection. In contrast, cyclin D3 was mainly present in the nucleus, with occasional localization in the cytoplasm, in non-infected cells. More importantly, at 10 hours post infection cyclin D3 was detected in the cytoplasm and at 24 hours post infection expression levels were much reduced.

To further study the consequences of influenza virus infection on cyclin D3 expression levels, we infected A549 cells for increasing time points and MOI and analyzed protein levels by western blotting. We found that level of expressed cyclin D3 is decreased upon infection and that this effect is dependent on both time of infection and MOI used. Those data suggest that cyclin D3 is either degraded or its expression down regulated upon influenza virus infection. Interestingly, protein levels of downstream effectors, namely cyclin dependent kinases CDK4 and 6, retinoblastoma protein RB, and cyclin E and A, were also diminished upon infection. By contrast, levels of the CDK inhibitors P15 and P16, which are upstream regulators of the G1-S transition, were unchanged.

Together, our data strongly suggest that influenza virus infection alters normal cell cycle progression by interfering with expression levels of cell cycle regulators. Most likely, the newly identified interaction between influenza M2 proton channel and cyclin D3 is involved in this process.

We will further investigate the effects of infection on the cell cycle progression by flow cytometry and decipher the molecular mechanism responsible for the down-regulation of cell cycle regulators in influenza infected cells. We will determine whether alteration of cell cycle progression is a strategy used by influenza virus to better replicate in host cells.

